# Comparison between a deep-learning and a pixel-based approach for the automated quantification of HIV target cells in foreskin tissue

**DOI:** 10.1038/s41598-024-52613-3

**Published:** 2024-01-23

**Authors:** Zhongtian Shao, Lane B. Buchanan, David Zuanazzi, Yazan N. Khan, Ali R. Khan, Jessica L. Prodger

**Affiliations:** 1 Department of Microbiology and Immunology, The University of Western Ontario, 1151 Richmond St, London, ON N6A 3K7 Canada; 2 Department of Medical Biophysics, The University of Western Ontario, 1151 Richmond St, London, ON N6A 3K7 Canada; 3 Department of Epidemiology and Biostatistics, The University of Western Ontario, 1151 Richmond St, London, ON N6A 3K7 Canada

**Keywords:** Machine learning, HIV infections, Image processing

## Abstract

The availability of target cells expressing the HIV receptors CD4 and CCR5 in genital tissue is a critical determinant of HIV susceptibility during sexual transmission. Quantification of immune cells in genital tissue is therefore an important outcome for studies on HIV susceptibility and prevention. Immunofluorescence microscopy allows for precise visualization of immune cells in mucosal tissues; however, this technique is limited in clinical studies by the lack of an accurate, unbiased, high-throughput image analysis method. Current pixel-based thresholding methods for cell counting struggle in tissue regions with high cell density and autofluorescence, both of which are common features in genital tissue. We describe a deep-learning approach using the publicly available StarDist method to count cells in immunofluorescence microscopy images of foreskin stained for nuclei, CD3, CD4, and CCR5. The accuracy of the model was comparable to manual counting (gold standard) and surpassed the capability of a previously described pixel-based cell counting method. We show that the performance of our deep-learning model is robust in tissue regions with high cell density and high autofluorescence. Moreover, we show that this deep-learning analysis method is both easy to implement and to adapt for the identification of other cell types in genital mucosal tissue.

## Introduction

Sexual transmission of HIV, which occurs at the anogenital mucosa, accounts for the majority of new HIV infections worldwide^1^. The availability of target cells bearing the HIV co-receptors CD4 and CCR5 in genital tissue is a critical determinant of productive infection^2–5^. The inner foreskin of the penis is enriched in HIV-susceptible T cell populations and other cell types in the foreskin such as macrophages, dendritic cells, and Langerhans cells, which can contribute to the spread of HIV by direct infection or by disseminating viral particles to susceptible T cells^6,7^. The genital mucosa is exposed to sexually transmitted pathogens, commensal bacteria, and exogenous materials (hygiene products, genital secretions, etc.) that can induce local inflammation and increase HIV susceptibility^8–13^. Thus, it is essential to directly quantify immune cells in genital mucosal tissue when testing new prevention modalities or determining best practices to prevent HIV transmission.

High-parameter flow cytometry can provide detailed information on the phenotype and activation state of immune cell subsets, but is ill-suited for measuring immune cell density in tissue^14^. Disruption of tissue into single cell suspensions for flow cytometry reduces the viability of sprawling cells, such as dendritic cells and Langerhans cells, and results in the loss of information on the spatial distribution of cells within tissue. Furthermore, tissue disruption may alter the expression of cell surface markers relevant to viral entry. Immunofluorescent (IF) imaging overcomes the limitations imposed by tissue disruption but introduces new challenges in quantification. Structural proteins and collagen fibers in genital mucosal tissue exhibit high levels of autofluorescence that can obscure positive staining and make cell quantification challenging^15^. Manual counting remains the gold standard but is laborious and variability and bias may be introduced by the operators. To overcome these issues, significant effort has recently been made to automate cell identification (cell segmentation) and quantification^16–20^.

Traditional automated cell segmentation workflows typically use a “bottom-up” pixel-based strategy where a threshold is applied so that every pixel is classified as positive staining or background. While this approach can be implemented in cases where cells are clearly separated, it struggles in areas that have densely packed cells or high autofluorescence^21^. Advances in deep learning have enabled “top-down” object-based approaches for cell segmentation where a prediction is made to identify individual cells with rough shape representations. Early attempts at using machine learning for cell segmentation utilized “bounding boxes” to create cell selections^22–24^. However, these approaches can be inaccurate in densely packed cell areas due to overlapping bounding boxes that impede cell detection. Recent studies have utilized better shape representations to replace bounding boxes for predictions. For instance, the deep learning segmentation method StarDist uses star-convex polygons to identify cells^25^. Star-convex polygons are highly suitable for approximating the shape of important HIV target cells since they can match the blob-like shape of CD4 + T cells while also maintaining enough flexibility to outline sprawling cells like dendritic cells.

Here, we describe the training and validation of a StarDist model to identify HIV target cells in foreskin tissue stained for CD3, CD4, CCR5, and nuclei. Specifically, we introduce a reusable workflow that can be used to train custom StarDist models. This workflow uses multi-channel IF microscopy images as input and is not limited by the tissue type or markers used for staining. The accuracy of this StarDist model was compared to manual counting and automated counting using a previously established pixel-based algorithm. The training workflow, training dataset and StarDist model are available in the public domain (https://github.com/prodgerlab/stardist).

## Materials and methods

### Ethics approval statement

This study utilizes foreskin samples from a randomized controlled trial exploring the effect of antimicrobial agents on the penile microbiota, immunology and HIV susceptibility of Ugandan men^26^. The randomized controlled trial protocol was reviewed and approved by the Ugandan Virus Research Institute’s Research and Ethics Committee (UVRI SEC), the Ugandan National Council for Science and Technology (UNCST), and the University of Toronto HIV Research Ethics Board. Written informed consent was obtained prior to participation in the study. Data and biological samples collected from study participants were labelled with a unique study identification number. All clinical events that were directly related to the study were referred to and managed by the UVRI-IAVI clinical team according to the Ugandan Ministry of Health guidelines. Participants in the study were not directly involved in the design of this study and did not directly assess the effect of the interventions. Participants did play a role in contributing to peer referral of potential participants. The Community Advisory Board for UVRI-IAVI meets quarterly and provides advice to the study team on study design and recruitment. The protocol for analysis of frozen tissue sections by IF (and correlation with data generated by collaborators) was approved by the Research Ethics Board at The University of Western Ontario.

### Immunofluorescence microscopy

Foreskin tissues used in this study (n = 40) were collected from adult males undergoing elective penile circumcision as part of a larger study on HIV prevention in Uganda^26^. Frozen tissue blocks were prepared in the Uganda Virus Research Institute-International AIDS Vaccine Initiative laboratory in Uganda immediately after circumcision and then shipped to the University of Western Ontario in Canada for immunofluorescent microscopy analysis. Frozen tissue blocks were sectioned at a thickness of 8 µm using a CM1850 cryostat (Leica, Wetzlar, Germany) and two sections from each tissue block were adhered to a glass slide for staining. Slides were stored in an air-tight box at − 80 °C for up to 1 month prior to staining. Slides were thawed and air-dried for 30 min, and tissue sections fixed by applying 100 µl of 3.7% formaldehyde in PIPES buffer (100 mM PIPES, 2 mM MgCl_2_, 1 mM EGTA in PBS, pH 6.8) for 5 min at room temperature. Slides were washed 3 times in 1 × PBS between all subsequent blocking and staining steps and all staining was performed in the dark at room temperature, unless otherwise stated. Each tissue section was blocked by applying 100 µl of a solution consisting of 10% normal donkey serum, 0.1% Triton X-100, and 0.01% sodium azide diluted in 1 × PBS (henceforth referred to as blocking solution) for 30 min. Each section was incubated with 100 µl of undiluted anti-human primary CD3 antibody (Clone: SP7) (Abcam, Cambridge, United Kingdom) for 1 h at 37 °C, followed by 30 min incubation with 100 µl of donkey anti-rabbit Alexa Fluor 488-conjugated secondary antibody (Polyclonal, diluted to 0.25% in blocking solution) (Abcam). Next, sections were each incubated with 100 µl of mouse anti-human CCR5 primary antibody (diluted to 5% in blocking buffer) (Monoclonal, a gift from Dr. Matthias Mack, University of Regensburg, Germany) for 1 h at 37 °C, followed by 30 min incubation with 100 µl of donkey anti-mouse Alexa Fluor 647-conjugated secondary antibody (Polyclonal, diluted to 0.25% in blocking solution) (Abcam). Finally, sections were each incubated with 100 µl goat anti-human CD4 primary antibody (Polyclonal, diluted to 5% in blocking buffer) (Abcam) overnight followed by 30 min incubation with 100 µl of donkey anti-goat Alexa Fluor 568-conjugated secondary antibody (Polyclonal, diluted to 0.25% in blocking buffer) (Abcam). Coverslips were mounted onto stained slides by applying 100 µl Fluoromount G mounting media with DAPI (Thermo Fisher Scientific, MA, USA) per slide. Slides were stored at 4 °C for up to one week prior to imaging. Tiled images for whole tissue sections were scanned with a DM5500B fluorescence microscope (Leica, Wetzlar, Germany) using the 20 × objective lens for CCR5 (Y5 filter set, referred to as far-red channel), CD4 (DSR filter set, referred to as red channel), CD3 (GFP filter set, referred to as green channel), and cell nuclei (CFP filter set, referred to as blue channel). Representative staining is depicted in Supplementary Fig. 1. Excitation and emission filters are listed in Table 1.

**Table 1:** List of microscope filters and their designations.

Fluorophore	Leica filter designation	Absorbance λ	Excitation filter*	Emission λ	Emission filter*	Emission colour
DAPI	CFP	358	436/20	461	480/40	Blue
Alexa fluor 488	GFP	494	425/60	517	480/LP	Green
Alexa fluor 546	DSR	556	545/30	573	620/60	Red
Alexa fluor 647	Y5	650	620/60	665	700/75	Far-Red

LP (long pass filter), λ (wavelength in nanometers).

*peak wavelength of light that passes through the filter and filter bandwidth.

### Manual counting of HIV target cells

All possible cell type combinations positive for CD3, CD4, CCR5, and nuclei were counted individually (i.e., all DAPI +, CD3 + /DAPI +, CD4 + /DAPI +, CCR5 + /DAPI +, CD3 + CD4 + /DAPI + CD3 + CCR5 + /DAPI +, CD4 + CCR5 + /DAPI +, and CD3 + CD4 + CCR5 + /DAPI +). Total cell count was determined by counting all positive stained nuclei (DAPI). Manual counting of each cell type was completed using composite images of nuclei staining and the marker(s) of interest only (e.g., manual counting of CD3 + cells was performed on a composite image created by merging the CD3 and DAPI channels). Counting was completed by a single trained lab member using the Cell Counter plugin for Fiji. A cell was considered positive for a marker when positive marker staining overlapped, surrounded, or was directly adjacent a positive nucleus (Supplementary Fig. 2).

### Pixel-based counting using Fiji and CellProfiler

Pixel-based cell counting was performed using an algorithm based on the open-source image analysis programs Fiji and CellProfiler^27–29^. Image processing functions in Fiji were used to pre-process images. Both Fiji and CellProfiler were used for thresholding and cell segmentation after pre-processing^27,28^. The epidermis and dermis underwent pre-processing, thresholding and segmentation using independent parameters, as the epidermis and dermis are very different in terms of cell density and level of autofluorescence.

Prior to pre-processing, full tissue section scans were split into the dermis and epidermis via manual tracing in Fiji and saved as separate files. The epidermis and dermis images were then split into individual channel images (CD3, CD4, CCR5, nuclei) by applying the “Stack to Images” function in Fiji. All individual channel images were pre-processed by applying the “Subtract Background”, “Brightness/Contrast”, and “Minimum Filter” functions successively. The “Subtract Background” and “Minimum Filer” functions were applied using parameters listed in Table 2; the “Auto” setting was used for used for all “Brightness/Contrast” adjustments.

**Table 2:** Input parameters for pre-processing workflow in Fiji for pixel-based cell counting.

Function	Input parameter	Epidermis				Dermis			
		Nuclei	CD3	CD4	CCR5	Nuclei	CD3	CD4	CCR5
Subtract background	Rolling ball radius	10	14	12	10	25	15	15	15
Minimum filter	Radius	0	2	1	0.5	2	2.5	1.5	1.5

Fiji was then used to apply threshold levels to pre-processed images and to select for positive staining. Pre-processed images were converted to 8-bit format and the “Adaptive Thresholding” plugin for ImageJ was used to threshold images^27^. The “Fill Holes”, “Despeckle”, “Erode”, and “Watershed” functions were then applied successively. Positive marker staining selection was completed by applying the “Analyze Particles” function with the options for “Display Results”, “Clear Results” and “Add to Manager” checked, and the “Circularity” setting set from 0.00 to 1.00 to detect all objects. The regions-of-interest (ROIs) generated after applying the “Analyze Particles” function were saved and overlayed with a black 8-bit image with the same dimensions as the image being analyzed to create a binary image representing positive marker signal. The number of ROIs for nuclei staining represents the total cell count of the image. Parameter values for each function are listed in Table 3.

**Table 3:** Input parameters for thresholding and segmentation in CellProfiler for pixel-based cell counting.

Function	Input parameter	Epidermis			Dermis			
		Nuclei	CD3	CD4	Nuclei	CD3	CD4	CCR5
Adaptive thresholding	Block size	100	100	100	100	100	100	100
Adaptive thresholding	Subtraction value	− 5	− 45	− 10	− 30	− 40	− 35	− 20
Analyze particles	Particle size	> 5	> 7	> 10	> 5	> 10	> 5	> 10

CellProfiler was then used to quantify cells of interest by determining the number of instances where each combination of positive marker staining overlapped with positive staining of nuclei. The binary black and white images of positive marker staining were imported into CellProfiler using the “ConvertImageToObjects” function. The “RelateObjects” function was then applied to determine positive marker staining for CD3 and/or CD4 and/or CCR5 that overlaps with positive nuclei staining^27,28^. Scripts for high-throughput pre-processing and automated counting of images are available in the public domain (https://github.com/prodgerlab/pixel-based-quantification).

### Annotation of images for StarDist model training in Labkit

A total of 40 fields-of-view (FOVs) were randomly generated from both inner and outer whole tissue section scans (600 × 600 µm, 1500 × 1500 pixels) for manual annotation for StarDist model training. Prior to annotation, all FOVs were visually assessed for exclusion of areas with tissue folding (due to improper adherence of tissue sections to glass slides during cryosectioning). Each FOV contained the full thickness of the epidermis (both the apical and basal edges of the epidermis clearly identifiable) and at least half of the full thickness of the dermis. All possible marker combinations (described above, in manual counting) were annotated individually in each FOV using the Labkit plugin (version 0.3.7) for Fiji^27,30^. All cells were then manually traced and assigned an individual label with the override option selected to prevent overlapping cells (Supplementary Fig. 2C,F). Completed annotation images were saved as separate tiff files. Each annotated set used for training included 1 raw 4-channel FOV image and 8 annotated images (considered as ground truth for training).

### StarDist model training

Training of the custom StarDist model was completed using a CUDA enabled Windows 10 workstation computer running a Window Subsystem for Linux with the Ubuntu (version 22.04) distribution of Linux installed. This workstation computer was equipped with an NVIDIA GeForce RTX 3090 graphics card to accelerate model training. The training environment was set up using the Mambaforge (version 0.24.0) distribution of Python (version 3.10.5) for Linux and TensorFlow (version 2.10.0) through a custom workflow created using Snakemake (version 7.12.0)^31^. The Snakemake workflow used to set up the training environment was adapted from instructions in the StarDist GitHub repository (https://github.com/stardist/stardist) that originally described set-up of a Python training environment using the Anaconda distribution of Python. Our full Snakemake workflow, which includes all scripts used for model training and export, is available in the public domain (https://github.com/prodgerlab/stardist).

The scripts used for model training and export were adapted from example Jupyter notebooks in the StarDist repository, which provided instruction for 2D (U-Net-based) StarDist model training. Adjustments were made so that IF microscopy images with 4 channels can be used as input. Image augmentation functions were adapted to include random flipping and intensity modification of training images, to improve the accuracy of the StarDist model without the need for additional training images and annotation. Training was completed with the n_rays parameter for Config2D set to 32 and all other parameters were left to their default value in accordance with the Jupyter notebook example. The full dataset used for training is available in the public domain (https://zenodo.org/record/8091914). Model training was performed for 400 epochs with 100 steps per epoch. Probability and overlap parameters for non-maximum suppression (NMS) post-processing were optimized and applied when using the model in the StarDist plugin (version 0.6.0) for Fiji^25,27^.

When using the model for prediction, images fed into the model were first normalized (percentile range 1.7 to 99.8). The model was then run with the probability/score threshold set to 0.50, overlap threshold set to 0.70, number of tiles set to 16, and boundary exclusion set to 2. Our trained StarDist model with optimized parameter pre-loaded is available in the public domain (https://zenodo.org/record/8091889).

### StarDist model validation

We compared the performance of our StarDist model against manual counting and pixel-based automated cell counting using 10 randomly selected FOV images (different from training FOVs), containing images from both inner and outer foreskin tissues. Validation FOVs were 600 × 600 µm (1500 × 1500 pixels) in size, excluded areas with tissue folding, and included the full thickness of the epidermis and at least half of the thickness of the dermis. Manual counting of the validation images was completed by a single trained lab member.

### StarDist model performance in areas of high autofluorescence or high cell density

Four image crops containing high autofluorescence (70 × 70 µm, 175 × 175 pixels) and four image crops containing high cell density (70 × 70 µm, 175 × 175 pixels) were created from each of the 10 validation FOVs (total n = 40 high autofluorescence and n = 40 high cell density image crops). Cells were quantified in image crops by manual counting, the StarDist model, and the pixel-based model.

### Statistical analyses

For StarDist model validation, the difference in FOV cell counts between manual counting and (i) the pixel-based model, and (ii) the StarDist model, was determined and statistical significance (p < 0.05) was assessed using a paired t test. Using manual counting as the gold standard, the total number of true positive, true negative, false positive, and false negative counts were determined for the pixel-based and StarDist models, and used to calculate each models sensitivity, precision, false negative rate, and false discovery rate.

Performance of the StarDist and pixel-based models in areas of high autofluorescence was assessed by quantifying the number of high autofluorescence image crops with at least one instance of autofluorescence inaccurately counted as a cell, compared to manual counting^25,27,29^. Performance in areas of high cell density was assessed by counting the number of high cell density image crops with at least one instance of a cell being either falsely merged or falsely split, compared to manual counting. Differences in Cell Merging, Cell Splitting, and Autofluorescence Misidentification between the Pixel-Based method and StarDist method was assess using Fisher’s exact tests.

All results are presented as mean ± SDs. Graphics were generated and statistical analyses performed using Prism 9 (GraphPad software, La Jolla, CA, USA).

## Results

### Accuracy of the custom StarDist model rapidly improves with additional training images

**Figure 1:**
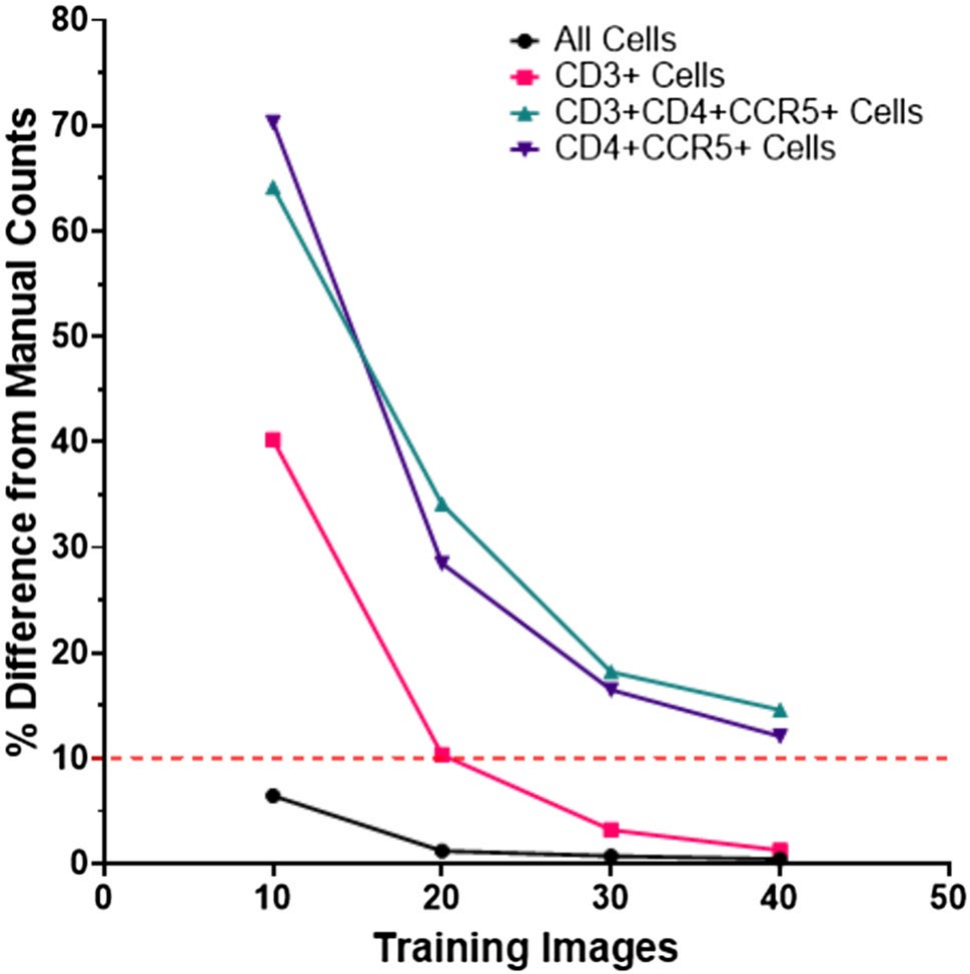
Training images rapidly improve the accuracy of a custom StarDist model used for the quantification of HIV target cells in foreskin tissue. Percent difference of automated counts generated by StarDist from manual counts for nuclei (all cells), CD3 + cells, CD4 + CCR5 + cells, and CD3 + CD4 + CCR5 + cells after training with 10 images, 20 images, 30 images, and 40 images. Automated counting was completed on 10 validation field-of-view images (600 × 600 µm) randomly generated from a set of 232 full foreskin tissue section scans stain for CD3, CD4, CCR5, and nuclei to identify HIV susceptible cells in the tissue.

The accuracy of the StarDist model in cell segmentation (compared to manual counting) was quantified for nuclei (all cells), CD3 + cells, CD4 + CCR5 + cells and CD3 + CD4 + CCR5 + cells after training with 10, 20, 30, and 40 images (Fig. 1). Pooled cell counts were generated from the 10 validation FOVs and percent difference from manual counts was used as a measure of accuracy. The accuracy of the StarDist model increased with each addition of 10 annotated images for training (Fig. 1). The percent difference of automated StarDist counts from manual counts decreased exponentially, with only small gains in accuracy made between 30 and 40 training FOVs (0.34% decrease for all cells (nuclei), 1.8% decrease for CD3 + cells, 5.2% decrease for CD4 + CCR5 + cells, and 4.6% decrease for CD3 + CD4 + CCR5 + cells).

### Automated HIV target cell segmentation using our StarDist model is comparable to manual counting and more accurate than pixel-based cell segmentation

**Figure 2:**
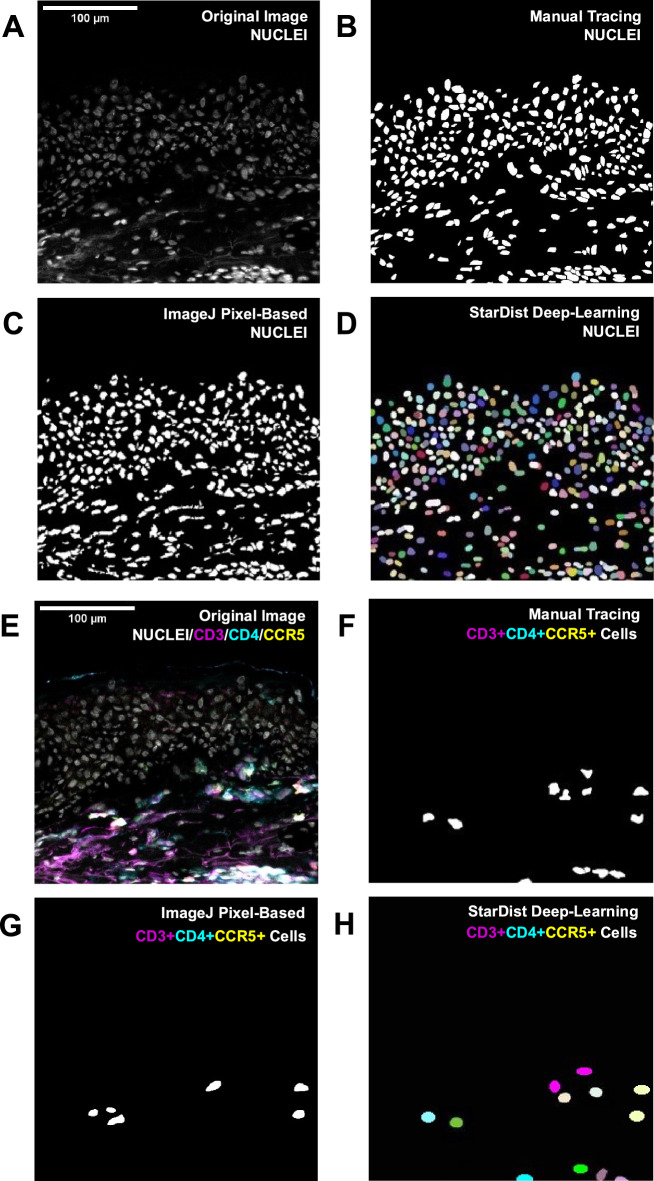
Automated segmentation of HIV target cells in foreskin tissue using pixel-based and StarDist models. (**A**,**E**) Representative image of (**A**) nuclei staining and (**E**) composite image of staining for CD3, CD4, CCR5, and nuclei were generated from a cropped image of a full tissue section scan of foreskin tissue imaged at 200 × total magnification. Visual representation of (**B**–**D**) counting of all positively stained nuclei and (**F**–**H**) CD3 + CD4 + CCR5 + cells in the representative image. Methods of cell segmentation presented include: (**B**,**F**) manual counting of cells, (**C**,**G**) automated counting using a pixel-based model, and (**D**,**H**) automated segmentation using a custom StarDist deep-learning model.

**Figure 3:**
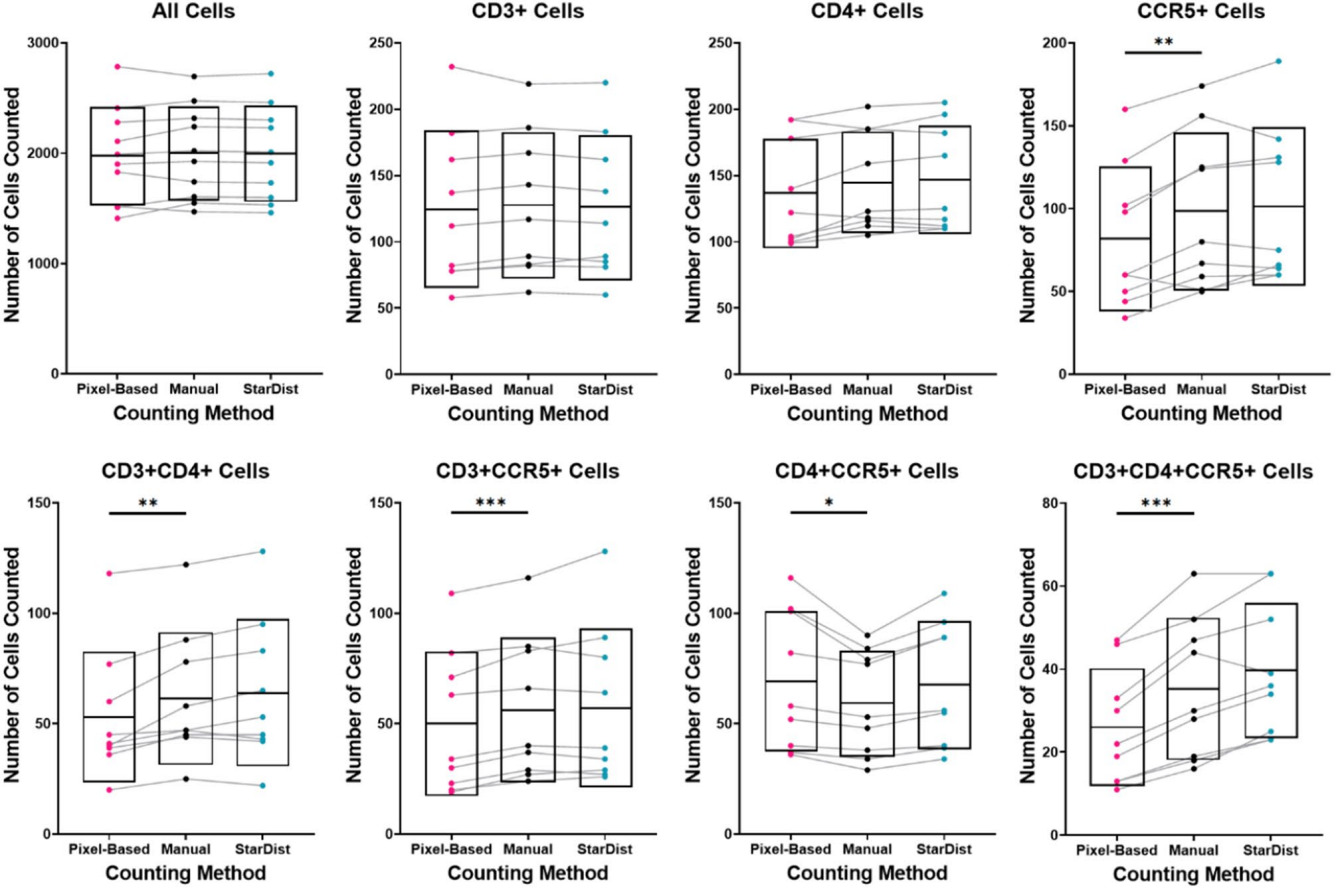
Comparison of manual cell counts with automated cell counts generated using a pixel-based model and the StarDist model. Cell counting was completed on 10 validation field-of-view images (600 × 600 µm) randomly generated from a set of 232 full section scans (imaged at 200 × total magnification) of foreskin tissue stained for CD3, CD4, CCR5, and nuclei. Manual counts are presented in the center (black dots), automated counts from the pixel-based model on the left (pink dots), and automated counts from the StarDist model on the right (cyan dots). Gray lines connect counts acquired by each method from the same validation FOV. The black center line and box represent the mean and SD. Differences between manual counts and the two automated counting methods was assess using two-tailed paired T-tests. Significant differences are indicated by asterisks (*p ≤ 0.05, **p ≤ 0.01, ***p ≤ 0.001).

To compare performance of our custom StarDist model to manual counting and a previously published pixel-based workflow, cells of interested were quantified in 10 FOVs (600 × 600 µm each) of foreskin tissue stained for CD3, CD4, CCR5, and nuclei (Fig. 2). Pairwise comparison of manual counts with counts generated by our StarDist model showed that total cell count and counts for all cell types of interest (CD3 + cells, CD4 + cells, CCR5 + cells, CD3 + CD4 + cells, CD3 + CCR5 + cells, CD4 + CCR5 + cells, CD3 + CD4 + CCR5 + cells) were not significantly different between the two methods (p > 0.05) (Fig. 3). Automated counts generated with the pixel-based model were not significantly different (p > 0.05) from manual counts for total cell count, CD3 + cells, and CD4 + cells, but differed significantly for all other cell types. Specifically, pixel-based model counts were significantly lower for CCR5 + cells (− 18.8%, p = 0.004), CD3 + CD4 + cells (− 16.5%, p = 0.006), CD3 + CCR5 + cells (− 12.8%, p = 0.0004), and CD3 + CD4 + CCR5 + cells (− 24.9%, p = 0.0005), and significantly higher for CD4 + CCR5 + cells (+ 14.2%, p = 0.025) (Fig. 3 and Supplementary Table 1).

Automated cell segmentation of HIV target cells in foreskin tissue using our custom StarDist model had higher sensitivity and precision than the pixel-based approach for all cell types. Sensitivity of the StarDist model was > 94.0% for all cell types analyzed and > 97.5% for CD3 + CCR5 + cell counts, CD4 + CCR5 + cell counts, and CD3 + CD4 + CCR5 + cell counts. Meanwhile the sensitivity of the pixel-based workflow was < 85.0% for all cell types except for counts for total cell count (93.6%) and CD3 + cells (91.1%). In particular, the sensitivity of the pixel-based model for CD3 + CD4 + CCR5 + cells was only 60.7%. Both the StarDist and the pixel-based models had > 90.0% precision for all cell types except for CD4 + CCR5 + cells and CD3 + CD4 + CCR5 + cells (StarDist: 87.9% and 85.2%, respectively; pixel-based: 69.8% and 78.0%, respectively) (Supplementary Table 1).

The StarDist model also had a lower false negative rate and lower false discovery rate than the pixel-based model all cell types. The false negative rate using the StarDist model was < 5.2% for all cell types, while the false negative rate for the pixel-based model ranged from 6.4% (total cell count) to 39.3% (CD3 + CD4 + CCR5 + cells). The false discovery rate for the pixel-based model was 1.7-fold (CD3 + cells) to 16.4-fold (CD3 + CD4 + CCR5 + cells) higher than the StarDist model. The false discover rate of both methods was < 10.0% for all cell types, except for CD4 + CCR5 + cells and CD3 + CD4 + CCR5 + cells (StarDist: 12.1% and 14.8%, respectively; for, pixel-based: 30.2% and 22.0%, respectively) (Supplementary Table 1).

### StarDist does not systematically over- or under-count in different tissue regions

**Figure 4:**
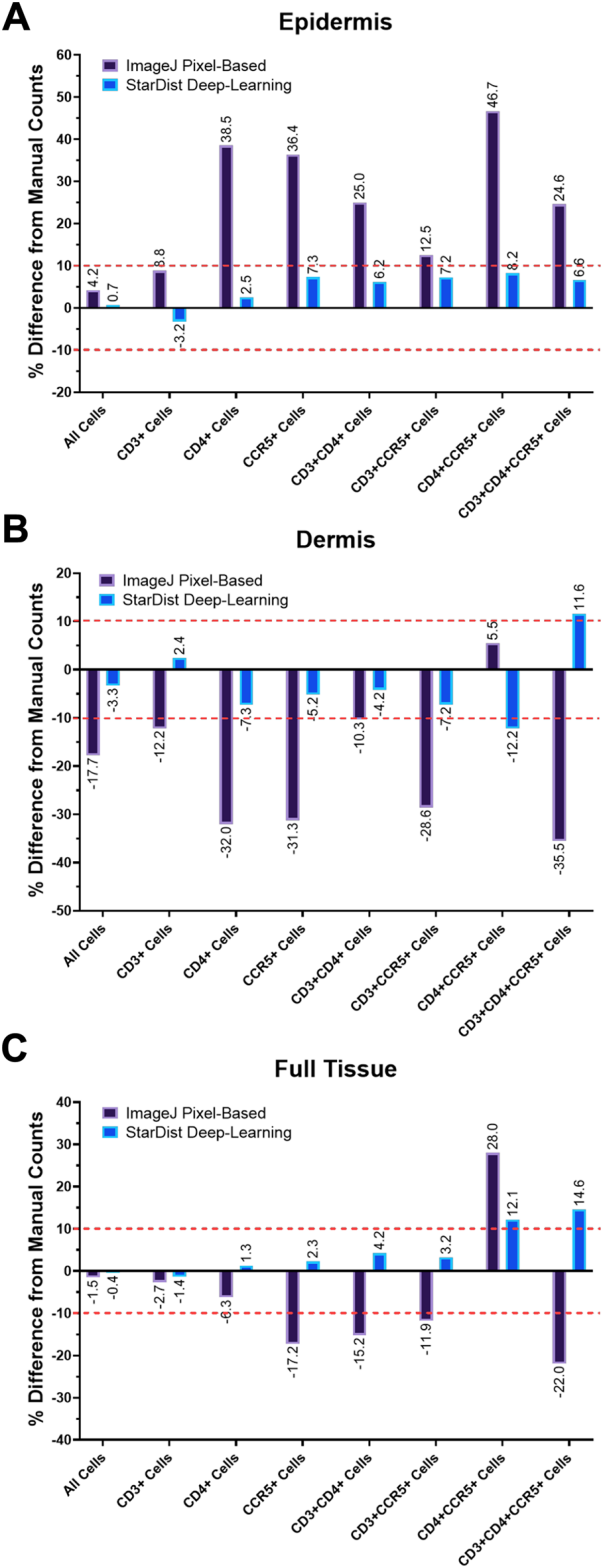
Automated cell segmentation using a pixel-based model systematically overcounts cells in the epidermis, and undercounts cells in the dermis. Cell counting was completed on 10 validation field-of-view images (600 × 600 µm) randomly selected from a set of 232 full section scans of foreskin tissues (inner and outer) stained for CD3, CD4, CCR5, and nuclei. Cell counting was completed on the full field-of-view images, and separately on the epidermis and dermis. The difference between automated counts and manual counts in (**A**) epidermis, (**B**) dermis, and (**C**) full tissue is displayed the percent difference from manual counts. Red dashed lines mark ± 10.0% difference from manual counts.

The epidermis and dermis regions of the foreskin are very different in terms of cell density and autofluorescence (Supplementary Fig. 1). To overcome this challenge, many pixel-based workflows (including the one presented here) process the epidermis and dermis/lamina propria regions separately, using different image analysis parameters, which increases processing time and labour. To determine if our StarDist model is also affected by systematic counting errors in different tissue regions, the 10 validation FOVs were manually split into epidermis and dermis regions and cell counts were obtained by the three methods (manual, pixel-based, StarDist) for each (Fig. 4).

In the epidermis (Fig. 4A), both automated methods (generally) overestimated the number of each cell type. This was much more dramatic for the pixel-based method; in particular, the percent difference from manual counts was > 25.0% for CD4 + cells, CCR5 + cells, CD3 + CD4 + cells, CD4 + CCR5 + cells, and CD3 + CD4 + CCR5 + cells. Percent differences between manual counting and the StarDist model were comparatively less, with undercounting of CD3 + cells (− 3.2%), and overcounting of all other cell types by < 10.0%. In the dermis (Fig. 4B), the pixel-based method displayed systematic undercounting. In particular, CD4 + cells, CCR5 + cells, CD3 + CCR5 + cells, and CD3 + CD4 + CCR5 + cells were all undercounted by > 20.0%. The StarDist model underestimated total cell count, CD4 + cells, CCR5 + cells, CD3 + CD4 + cells and CD3 + CCR5 + cells, all by < 10.0%, and overcounted CD3 + cells (2.4%), CD4 + CCR5 + cells (12.2%), and CD3 + CD4 + CCR5 + cells (11.6%). Systematic overcounting in the epidermis and undercounting in the dermis by the pixel-based method resulted in misleadingly low overall percent differences from manual counts with both regions considered in full tissues (Fig. 4C).

### Automated cell segmentation with StarDist is robust in areas with high cell density or high autofluorescence

**Figure 5:**
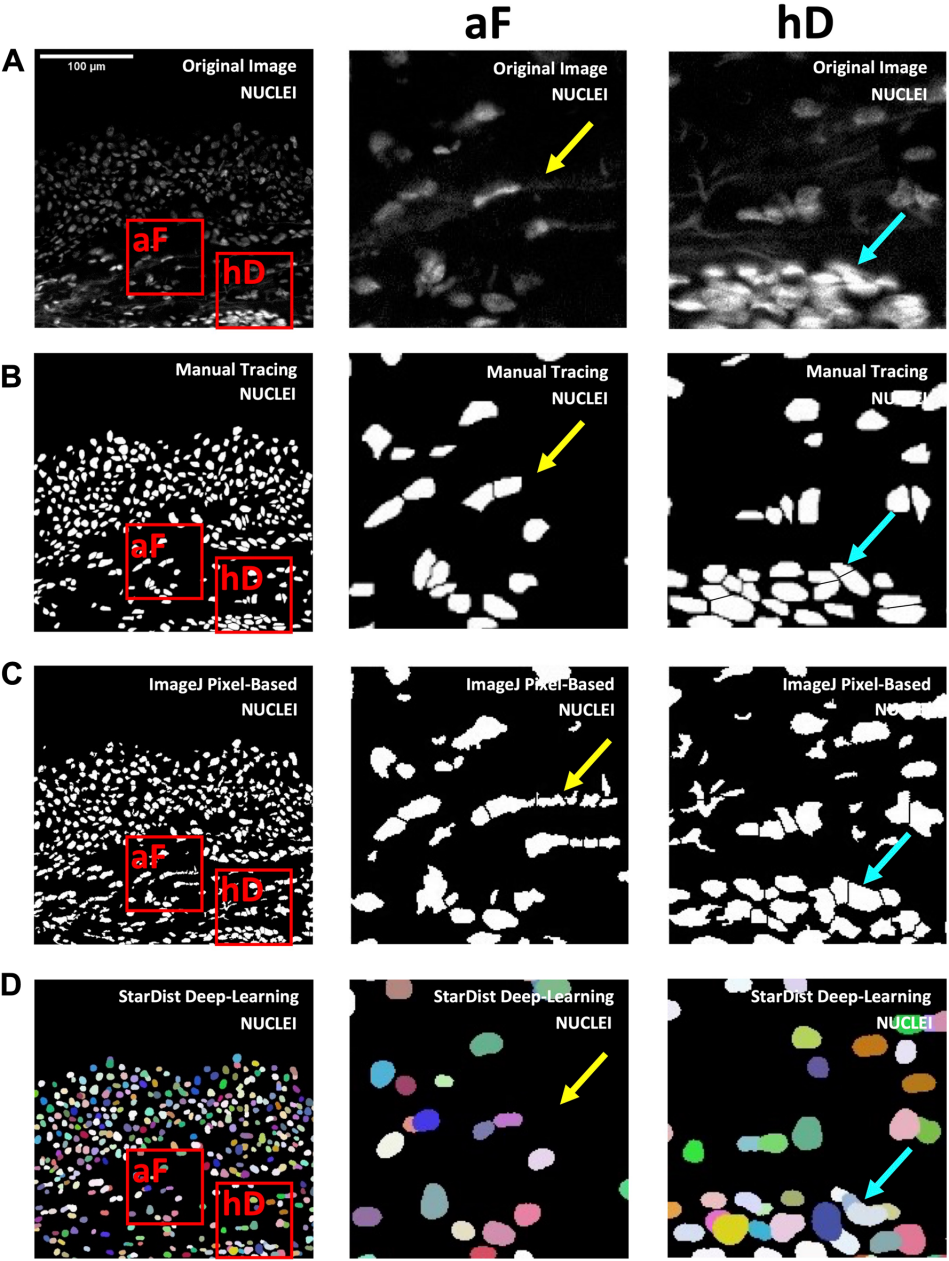
Comparison of StarDist and pixel-based model nucleus segmentation in tissue regions with high cell density or high autofluorescence. (**A**) Representative validation field-of-view of immunofluorescence microscopy image of foreskin tissue stained with DAPI (nuclei), showing examples of image crops (70 × 70 µm) with either (**aF**) high autofluorescence or (**hD**) high cell density (imaged at 200 × total magnification). Subsequent panels show the same FOV/image crops with nuclei segmentation by (**B**) manual tracing (gold standard), (**C**) the pixel-based model, and (**D**) the StarDist model. Yellow arrows point to areas with autofluorescent collagen fibers and cyan arrows point to areas with tightly packed cells.

Automated cell quantification methods often perform poorly in areas with high cell density (cell splitting) or high autofluorescence (false positives), both of which are found in foreskin and other mucosal/epithelial tissues. To assess performance specifically in areas of high cell density and autofluorescence, image crops (70 × 70 µm) were selected from each FOV (Fig. 5A) based on having high autofluorescence (4 crops per FOV, n = 40, denoted by “aF”) or high cell density (4 crops per FOV, n = 40, denoted by “hD”). The performance of manual counts (Fig. 5B), pixel-based methods (Fig. 5C), and StarDist model (Fig. 5D) was assessed for each crop within the same tissue region. For high autofluorescence image crops, the number of images where autofluorescent collagen fibers were misidentified as cells (compared to manual counting, Fig. 5B,aF) was counted for the pixel-based (Fig. 5C,aF) and StarDist models (Fig. 5D,aF). For high cell density image crops, the number of images where cell merging or splitting occurred during cell segmentation (compared to manual counting, Fig. 5B,hD) were counted for the pixel-based (Fig. 5C,hD) and StarDist models (Fig. 5D,hD).

In high cell density image crops, the pixel-based model merged cells significantly more frequently than the StarDist model in all cell types except CD3 + CD4 + cells, CD4 + CCR5 + cells, and CD3 + CD4 + CCR5 + cells, for which minimal merging was observed in both models (Supplementary Table 2). The pixel-based model also split cells more frequently, splitting nuclei, CD3 + cells, CD4 + cells, and CCR5 + cells significantly more frequently than the StarDist model. In general, the number of image crops where cell merging occurred was twofold (CD4 + CCR5 + cells) to sevenfold (CD4 + cells) higher when using the pixel-based model compared to the StarDist model. Image crops with cell merging or splitting were most common for the segmentation of nuclei, likely due to the high number of nuclei per crop.

Misidentification of autofluorescence as cells occurred significantly more frequently when using the pixel-based method, with misidentification observed for all cell types except CD4 + CCR5 + cells and CD3 + CD4 + CCR5 + cells (Supplementary Table 2). For both the pixel-based approach and StarDist, misidentification of autofluorescence as cells was most prevalent for CD3 + cell segmentation (35/40 image crops for pixel-based, 5/40 image crops for StarDist) (Supplementary Table 2).

## Discussion

In this report, we described the development and validation of a deep-learning approach to quantify HIV target cells in foreskin tissues. Using the Snakemake workflow management system and the StarDist plugin for Fiji, we trained a custom StarDist deep-learning model that can be used to identify HIV target cells in multi-channel immunofluorescent microscopy images of foreskin tissue^25,27,31^. The accuracy of this deep-learning model was compared to manual counting and a conventional automated cell segmentation algorithm which utilizes a pixel-based approach^29^. The reliability of the deep-learning model was also further tested in tissue regions with high cell density or high autofluorescence.

Immunofluorescent microscopy is an excellent tool for understanding the spatial distribution of immune cells in genital mucosal tissues, however, quantification of cells in IF microscopy images can be difficult. The subjective and labour-intensive nature of manual counting makes this method impractical for clinical studies. Automated approaches for cell segmentation often use a pixel-based workflow, however, the reliability of this method is poor in regions with high cell density and high autofluorescence^17,32^.

Our deep-learning approach to quantify HIV target cells in the foreskin can replace conventional pixel-based methods for immune cell quantification in genital tissues and can be easily adapted to detect of a wide variety of immune cell subsets in different types of genital mucosal tissue. Our approach utilizes multi-channel IF microscopy images as input and enables the detection of multiple cell types (based on combinations of markers) simultaneously. We used StarDist and its plugin for Fiji as the backbone for the development because of its accessibility. StarDist is open source and can easily be incorporated into existing image analysis workflows^25,27^. Plugins that allow StarDist models to be run without any programming knowledge are available for popular open-source software such as ImageJ/Fiji, Napari, QuPath, Icy, and KNIME^27,33–35^. Results from StarDist models are also highly reproducible since trained StarDist models and training datasets can be easily shared.

Our custom StarDist model exceeded conventional pixel-based cell segmentation in performance metrics including percent difference from manual counts, sensitivity, precision, false negative rate, and false discovery rate. Most notably, our custom StarDist model achieved over 94.0% sensitivity and over 85.0% precision for all cell types examined. There were no significant differences between StarDist counts and manual counts for all cell types examined. The percent difference of StarDist counts from manual counts was also less than 5.0% for all cell types except for CD4 + CCR5 + cells (11.3%) and CD3 + CD4 + CCR5 + cells (13.6%). These cell types were the rarest among all cell types we examined, thus fewer instances were available for training compared to other cell types. Detection of these cell types is also more complex, as image information from 3 channels are used to detect CD4 + CCR5 + cells and image information from 4 channels are used to detect CD3 + CD4 + CCR5 + cells. While the count accuracy for these cell types is likely to increase with more training, CD4 + CCR5 + cells are less likely to be T cells and more likely to be dendritic or Langerhans cells. A limitation of our StarDist model is that the star-convex polygons shape approximation strategy used may struggle to approximate the irregular shape of sprawling cells^36^. Recent work by Mandal et al. used planar parametric spline curves for cell segmentation in a method called SplineDist^37^. This method was shown to be capable of being incorporated into the StarDist architecture to accurately capture non-star-convex objects.

Cell detection using our StarDist model was robust in challenging tissue areas with high cell density or high levels of autofluorescence. Performance of conventional automated cell segmentation can struggle in both the epidermis and dermis region of foreskin tissue for different reasons. In the epidermis, thresholding can be difficult due to high cell density. In the dermis, thresholding can be difficult due to the high abundance of autofluorescent collagen fibers. In contrast to conventional automated cell segmentation that relies on thresholding, the StarDist model did not systematically overcount or undercount in the epidermis or dermis, and detected cells with high accuracy in areas of high cell density or high autofluorescence. Based on these results, we conclude that the performance of our StarDist model is comparable to manual counting and StarDist is superior to conventional pixel-based approaches for HIV target cell quantification.

A limitation of this study is that we did not validate performance of the StarDist model in other tissue types or on other microscopes. It is likely that additional training, using the Snakemake framework^25,27,31^, would be necessary to use this model, for example, on cervical tissues or at other sites. Furthermore, our StarDist model is trained using foreskin tissue stained for CD3, CD4, CCR5, and nuclei. While this marker set allows for quantification of all cells bearing the HIV co-receptors (CD4 and CCR5), additional training would be required to analyze tissues stained for different markers. Shared repositories of training sets with various tissue types may be used in the future to increase the generalizability of our StarDist model, without the requirement of new tissue collection from varied mucosae and denovo staining. However, existing repositories mainly contain IF images of only nuclei or histology images. Improving the diversity of training images in these repositories would greatly improve the adoptability of deep-learning methods for cell segmentation^38,39^.

An additional limitation of discrete cell segmentation methods, including our StarDist model, is that positive staining is only quantified if it is associated with a nucleus. The appendages of sprawling HIV target cells such as Langerhans cells and dendritic cells can often span through multiple tissue sections, resulting in cell membrane area that is positively stained for CD4 and CCR5 but unassociated with nuclei. Cell segmentation methods do not account for these appendages, and thus ignores some membrane surface area that may bind HIV virions. While pixel-based methods for image analysis are problematic for discrete cell segmentation, they are good for measuring the total amount of tissue area that is positively stained for a particular marker. Thus, pairing StarDist HIV target cell counting results with results from pixel-based quantification of CD4 + CCR5 + tissue area could be beneficial in terms of gaining a more holistic understanding of HIV susceptibility in foreskin tissue.

To our knowledge, this study is the first to describe a deep learning method for the quantification of HIV target cells in microscopy images of mucosal tissue. This is a highly relevant outcome for clinical trials aiming to reduce HIV susceptibility, as the availability of susceptible cells in the mucosa is directly linked to the likelihood that sexual exposure will lead to infection^40–42^. This is an important outcome not only for trials aimed at reducing target cell availability, but all trials of mucosally applied products, to ensure there are no off-target inflammatory effects that would unintentionally increase HIV-susceptibility^43,44^. Previous studies have extensively described deep-learning methods for segmentation of CD3 + cells but these methods focus on histology images^45,46^. Existing methods for deep-learning cell segmentation in IF microscopy images are not designed to enable simultaneous quantification of multiple cell types, limiting their utility to study the distribution and composition of immune cells. Implementation of these models also require a higher-level understanding of convolutional neural networks and software packages related to neural network construction^47,48^.

In conclusion, this StarDist deep learning approach is an accurate and time saving approach that can be used in a high-throughput manner to quantify immune cells in full mucosal tissue section scans. This model is more robust than conventional pixel-based methods in regions of high cell density and autofluorescence, and accurately quantifies cells in both the epidermal and dermal regions of the foreskin. This model can be easily applied and incorporated into existing ImageJ workflows, making it an appropriate replacement for conventional pixel-based methods for automated HIV target cell quantification that have previously been used in other studies. The high accuracy of this model enables high-throughput processing of samples from large patient cohorts and provides feasibility for the use of immunofluorescence imaging as reliable method for measuring HIV susceptibility in clinical trials.

### Supplementary Information

Supplementary Information.

## Data Availability

The sample dataset acquired from training images can be accessed via the following public domain: (https://zenodo.org/record/8091914). Necessary scripts to perform the StarDist model training and validation are available in another public domain: (https://github.com/prodgerlab/stardist).

## References

[1] Ward H, Rönn M (2010). The contribution of STIs to the sexual transmission of HIV. Curr. Opin. HIV AIDS.

[2] Lemos MP, Lama JR, Karuna ST, Fong Y, Montano SM, Ganoza C, Gottardo R, Sanchez J, McElrath MJ (2014). The inner foreskin of healthy males at risk of HIV infection harbors epithelial CD4+ CCR5+ cells and has features of an inflamed epidermal barrier. PLoS One.

[3] McCoombe SG, Short RV (2006). Potential HIV-1 target cells in the human penis. AIDS.

[4] Reis Machado J, da Silva MV, Cavellani CL, dos Reis MA, Monteiro MLGR, Teixeira VPA, Miranda Corrêa RR (2014). Mucosal immunity in the female genital tract. HIV/AIDS Biomed. Res. Int..

[5] Shen R, Richter HE, Smith PD (2014). Interactions between HIV-1 and mucosal cells in the female reproductive tract. Am. J. Reprod. Immunol..

[6] Cavrois M, Neidleman J, Kreisberg JF, Greene WC (2007). In vitro derived dendritic cells trans-infect CD4 T cells primarily with surface-bound HIV-1 virions. PLoS Pathog..

[7] Kijewski SD, Gummuluru S (2015). A mechanistic overview of dendritic cell-mediated HIV-1 trans infection: The story so far. Fut. Virol..

[8] Boily M-C, Baggaley RF, Wang L, Masse B, White RG, Hayes RJ, Alary M (2009). Heterosexual risk of HIV-1 infection per sexual act: Systematic review and meta-analysis of observational studies. Lancet Infect Dis..

[9] Liu CM, Prodger JL, Tobian AAR, Abraham AG, Kigozi G, Hungate BA, Aziz M, Nalugoda F, Sariya S, Serwadda D, Kaul R, Gray RH, Price LB (2017). Penile anaerobic dysbiosis as a risk factor for HIV infection. MBio.

[10] Liu CM, Hungate BA, Tobian AAR, Ravel J, Prodger JL, Serwadda D, Kigozi G, Galiwango RM, Nalugoda F, Keim P, Wawer MJ, Price LB, Gray RH (2015). Penile microbiota and female partner bacterial vaginosis in Rakai, Uganda. MBio.

[11] Powers KA, Poole C, Pettifor AE, Cohen MS (2008). Rethinking the heterosexual infectivity of HIV-1: A systematic review and meta-analysis. Lancet Infect Dis..

[12] Prodger JL, Gray RH, Shannon B, Shahabi K, Kong X, Grabowski K, Kigozi G, Nalugoda F, Serwadda D, Wawer MJ, Reynolds SJ, Liu CM, Tobian AAR, Kaul R (2016). Chemokine levels in the penile coronal sulcus correlate with HIV-1 acquisition and are reduced by male circumcision in Rakai, Uganda. PLOS Pathogens.

[13] Prodger JL, Abraham AG, Tobian AAR, Park DE, Aziz M, Roach K, Gray RH, Buchanan L, Kigozi G, Galiwango RM, Ssekasanvu J, Nnamutete J, Kagaayi J, Kaul R, Liu CM (2021). Penile bacteria associated with HIV seroconversion, inflammation, and immune cells. JCI Insight.

[14] Aghaeepour N, Chattopadhyay PK, Ganesan A, O’Neill K, Zare H, Jalali A, Hoos HH, Roederer M, Brinkman RR (2012). Early immunologic correlates of HIV protection can be identified from computational analysis of complex multivariate T-cell flow cytometry assays*. Bioinformatics.

[15] Dinh MH, Anderson MR, McRaven MD, Cianci GC, McCoombe SG, Kelley ZL, Gioia CJ, Fought AJ, Rademaker AW, Veazey RS, Hope TJ (2015). Visualization of HIV-1 interactions with penile and foreskin epithelia: Clues for female-to-male HIV transmission. PLoS Pathog..

[16] Carpenter AE, Jones TR, Lamprecht MR, Clarke C, Kang IH, Friman O, Guertin DA, Chang JH, Lindquist RA, Moffat J, Golland P, Sabatini DM (2006). Cell Profiler: Image analysis software for identifying and quantifying cell phenotypes. Genome Biol..

[17] O’Brien J, Hayder H, Peng C (2016). Automated quantification and analysis of cell counting procedures using ImageJ plugins. J. Vis. Exp..

[18] Grishagin IV (2015). Automatic cell counting with ImageJ. Anal. Biochem..

[19] Falk T, Mai D, Bensch R, Çiçek Ö, Abdulkadir A, Marrakchi Y, Böhm A, Deubner J, Jäckel Z, Seiwald K, Dovzhenko A, Tietz O, Dal Bosco C, Walsh S, Saltukoglu D, Tay TL, Prinz M, Palme K, Simons M, Diester I, Brox T, Ronneberger O (2019). U-Net: Deep learning for cell counting, detection, and morphometry. Nat. Methods.

[20] Xie W, Noble JA, Zisserman A (2018). Microscopy cell counting and detection with fully convolutional regression networks. Comput. Methods Biomech. Biomed. Eng. Imaging Vis..

[21] A.J. Walsh, M.C. Skala, An automated image processing routine for segmentation of cell cytoplasms in high-resolution autofluorescence images, in: Multiphoton Microscopy in the Biomedical Sciences XIV, SPIE, 2014: pp. 161–166. 10.1117/12.2040644.

[22] Lee SMW, Shaw A, Simpson JL, Uminsky D, Garratt LW (2021). Differential cell counts using center-point networks achieves human-level accuracy and efficiency over segmentation. Sci. Rep..

[23] Yang L, Ghosh RP, Franklin JM, Chen S, You C, Narayan RR, Melcher ML, Liphardt JT (2020). NuSeT: A deep learning tool for reliably separating and analyzing crowded cells. PLoS Comput. Biol..

[24] Chen J, Zhang B (2021). Segmentation of overlapping cervical cells with mask region convolutional neural network. Comput. Math. Methods Med..

[25] Schmidt U, Weigert M, Broaddus C, Myers G (2018). Cell Detection with Star-Convex Polygons.

[26] Galiwango RM, Bagaya B, Mpendo J, Joag V, Okech B, Nanvubya A, Ssetaala A, Muwanga M, Kaul R (2019). Protocol for a randomized clinical trial exploring the effect of antimicrobial agents on the penile microbiota, immunology and HIV susceptibility of Ugandan men. Trials.

[27] Schindelin J, Arganda-Carreras I, Frise E, Kaynig V, Longair M, Pietzsch T, Preibisch S, Rueden C, Saalfeld S, Schmid B, Tinevez J-Y, White DJ, Hartenstein V, Eliceiri K, Tomancak P, Cardona A (2012). Fiji: An open-source platform for biological-image analysis. Nat. Methods.

[28] Stirling DR, Swain-Bowden MJ, Lucas AM, Carpenter AE, Cimini BA, Goodman A (2021). Cell Profiler 4: Improvements in speed, utility and usability. BMC Bioinform..

[29] Buchanan LB, Shao Z, Jiang YC, Lai A, Hope TJ, Carias AM, Prodger JL (2022). Quantitative immunofluorescent imaging of immune cells in mucosal tissues. Methods Mol. Biol..

[30] Arzt M, Deschamps J, Schmied C, Pietzsch T, Schmidt D, Tomancak P, Haase R, Jug F (2022). LABKIT: Labeling and segmentation toolkit for big image data. Front. Comput. Sci..

[31] Mölder F, Jablonski KP, Letcher B, Hall MB, Tomkins-Tinch CH, Sochat V, Forster J, Lee S, Twardziok SO, Kanitz A, Wilm A, Holtgrewe M, Rahmann S, Nahnsen S, Köster J (2021). Sustainable data analysis with Snakemake. F1000Research.

[32] Kesler B, Li G, Thiemicke A, Venkat R, Neuert G (2019). Automated cell boundary and 3D nuclear segmentation of cells in suspension. Sci. Rep..

[33] Bankhead P, Loughrey MB, Fernández JA, Dombrowski Y, McArt DG, Dunne PD, McQuaid S, Gray RT, Murray LJ, Coleman HG, James JA, Salto-Tellez M, Hamilton PW (2017). QuPath: Open source software for digital pathology image analysis. Sci. Rep..

[34] Sofroniew N, Lambert T, Evans K, Nunez-Iglesias J, Bokota G, Winston P, Peña-Castellanos G, Yamauchi K, Bussonnier M, Doncila Pop D, Can Solak A, Liu Z, Wadhwa P, Burt A, Buckley G, Sweet A, Migas L, Hilsenstein V, Gaifas L, Bragantini J, Rodríguez-Guerra J, Muñoz H, Freeman J, Boone P, Lowe A, Gohlke C, Royer L, Pierré A, Har-Gil H, McGovern A (2022). Zenodo.

[35] de Chaumont F, Dallongeville S, Chenouard N, Hervé N, Pop S, Provoost T, Meas-Yedid V, Pankajakshan P, Lecomte T, Le Montagner Y, Lagache T, Dufour A, Olivo-Marin J-C (2012). Icy: An open bioimage informatics platform for extended reproducible research. Nat. Methods.

[36] Korfhage N, Mühling M, Ringshandl S, Becker A, Schmeck B, Freisleben B (2020). Detection and segmentation of morphologically complex eukaryotic cells in fluorescence microscopy images via feature pyramid fusion. PLOS Computat. Biol..

[37] S. Mandal, V. Uhlmann, Splinedist: Automated Cell Segmentation With Spline Curves, in: 2021 IEEE 18th International Symposium on Biomedical Imaging (ISBI). pp. 1082–1086. 10.1109/ISBI48211.2021.9433928. (2021).

[38] Kromp F, Bozsaky E, Rifatbegovic F, Fischer L, Ambros M, Berneder M, Weiss T, Lazic D, Dörr W, Hanbury A, Beiske K, Ambros PF, Ambros IM, Taschner-Mandl S (2020). An annotated fluorescence image dataset for training nuclear segmentation methods. Sci. Data.

[39] Wagner M, Reinke S, Hänsel R, Klapper W, Braumann U-D (2020). An image dataset related to automated macrophage detection in immunostained lymphoma tissue samples. Gigascience.

[40] Carnathan DG, Wetzel KS, Yu J, Lee ST, Johnson BA, Paiardini M, Yan J, Morrow MP, Sardesai NY, Weiner DB, Ertl HJ, Silvestri G (2015). Activated CD4+CCR5+ T cells in the rectum predict increased SIV acquisition in S*IVGag/Tat-vaccinated rhesus macaques*. Proc. Natl. Acad. Sci..

[41] Chahroudi A, Cartwright E, Lee ST, Mavinger M, Carnathan DG, Lawson B, Carnathan PM, Hashempoor T, Murphy MK, Meeker T, Ehnert S, Sounder C, Else JG, Cohen J, Collman RG, Vanderford TH, Permar SR, Derdeyn CA, Villinger F, Silvestri G (2014). Target cell availability, rather than breast milk factors, dictates mother-to-infant transmission of SIV in sooty mangabeys and rhesus macaques. PLoS Pathog..

[42] Prodger J, Kaul R (2017). The biology of how circumcision reduces HIV susceptibility: broader implications for the prevention field. AIDS Res. Ther..

[43] Lajoie J, Birse K, Mwangi L, Chen Y, Cheruiyot J, Akolo M, Mungai J, Boily-Larouche G, Romas L, Mutch S, Kimani M, Oyugi J, Ho EA, Burgener A, Kimani J, Fowke KR (2018). Using safe, affordable and accessible non-steroidal anti-inflammatory drugs to reduce the number of HIV target cells in the blood and at the female genital tract. J. Int. AIDS Soc..

[44] Damme LV, Ramjee G, Alart M, Vuylsteke B, Chanderying V, Rees H, Sirivongrangson P, Mukenge-Tshibaka L, Ettègne-Traoré V, Uaheowitchai C, Karim SA, Mâsse B, Perriëns J, Laga M (2002). COL-1492 study group, effectiveness of COL-1492, a nonoxynol-9 vaginal gel, on HIV-1 transmission in female sex workers: A randomised controlled trial. Lancet.

[45] Zafar MM, Rauf Z, Sohail A, Khan AR, Obaidullah M, Khan SH, Lee YS, Khan A (2022). Detection of tumour infiltrating lymphocytes in CD3 and CD8 stained histopathological images using a two-phase deep CNN. Photodiagnosis Photodyn. Ther..

[46] Negahbani F, Sabzi R, Pakniyat Jahromi B, Firouzabadi D, Movahedi F, Kohandel Shirazi M, Majidi S, Dehghanian A (2021). PathoNet introduced as a deep neural network backend for evaluation of Ki-67 and tumor-infiltrating lymphocytes in breast cancer. Sci. Rep..

[47] Sadanandan SK, Ranefall P, Le Guyader S, Wählby C (2017). Automated training of deep convolutional neural networks for cell segmentation. Sci. Rep..

[48] Lee MY, Bedia JS, Bhate SS, Barlow GL, Phillips D, Fantl WJ, Nolan GP, Schürch CM (2022). Cell Seg: A robust, pre-trained nucleus segmentation and pixel quantification software for highly multiplexed fluorescence images. BMC Bioinform..

