# Supplementary material for: Comparison between a deep-learning and a pixel-based approach for the automated quantification of HIV target cells in foreskin tissue
Source: Sci Rep. 2024 Jan 23;14:1985. doi: 10.1038/s41598-024-52613-3 (PMC10806185; doi:10.1038/s41598-024-52613-3)
Supplement: Supplementary file 1 — Supplementary Information. [file 41598_2024_52613_MOESM1_ESM.pdf]

Comparison between a deep-learning and a pixel-based approach for the automated quantification of HIV target cells in foreskin tissue.

Zhongtian Shao[1], Lane B. Buchanan[1], David Zuanazzi[1], Yazan N. Khan[1], Ali R. Khan[2] and Jessica L. Prodger[*1,3]

Supplementary Table 1: Foreskin cell counts from n=10 immunofluorescence microscopy fields-of view and performance metrics of the pixel-based and StarDist models, compared to manual counting.

		Cell Types							
		**All Cells**	**CD3+ Cells**	**CD4+ Cells**	**CCR5+ Cells**	**CD3+ CD4+ Cells**	**CD3+ CCR5+ Cells**	**CD4+ CCR5+ Cells**	**CD3+ CD4+ CCR5+ Cells**
**Total Counts***	Manual	20037	1697	1843	1231	939	800	792	542
	Pixel-Based	19736	1651	1726	1019	796	704	1013	422
	StarDist	19956	1673	1866	1259	978	825	887	621
**% Difference from Manual Counts**	Pixel-Based	1.51%	2.75%	6.56%	18.84%	16.48%	12.77%	14.19%	24.90%
	StarDist	0.41%	1.42%	1.24%	2.25%	4.07%	3.08%	11.32%	13.59%
**% Change from Manual Counts**	Pixel-Based	-1.50%	-2.71%	-6.35%	-17.22%	-15.23%	-12.00%	15.28%	-22.14%
	StarDist	-0.40%	-1.41%	1.25%	2.27%	4.15%	3.13%	11.99%	14.58%
**True Positives (TP)**	Pixel-Based	18750	1546	1554	918	754	639	637	329
	StarDist	19720	1609	1780	1185	903	788	780	529
**False Positive (FP)**	Pixel-Based	986	105	172	101	42	65	276	93
	StarDist	236	64	86	74	75	37	107	92
**False Negative (FN)**	Pixel-Based	1287	151	289	313	185	161	155	213
	StarDist	317	88	63	46	36	12	12	13
**Sensitivity/ True Positive Rate (TPR)**	Pixel-Based	93.58%	91.10%	84.32%	74.57%	80.30%	79.88%	80.43%	60.70%
	StarDist	98.42%	94.81%	96.58%	96.26%	96.17%	98.50%	98.48%	97.60%
**Precision/ Positive predictive value (PPV)**	Pixel-Based	95.00%	93.64%	90.03%	90.09%	94.72%	90.77%	69.77%	77.96%
	StarDist	98.82%	96.17%	95.39%	94.12%	92.33%	95.52%	87.94%	85.19%
**False negative rate (FNR)**	Pixel-Based	6.42%	8.90%	15.68%	25.43%	19.70%	20.13%	19.57%	39.30%
	StarDist	1.58%	5.19%	3.42%	3.74%	3.83%	1.50%	1.52%	2.40%
**False discovery rate (FDR)**	Pixel-Based	5.00%	6.36%	9.97%	9.91%	5.28%	9.23%	30.23%	22.04%
	StarDist	1.18%	3.83%	4.61%	5.88%	7.67%	4.48%	12.06%	14.81%

*Generated from 10 FOV images (600x600 μm) of foreskin tissue stained for CD3, CD4, CCR5, and nuclei.

Supplementary Table 2: Number of image crops with incorrect cell segmentation (compared to manual counting) in regions of high cell density or high autofluorescence.

	Image Crops with High Cell Density (n=40)						Image Crops with High Autofluorescence (n=40)		
	Cell Merging*			Cell Splitting*			Autofluorescence Misidentification**		
Cell Types	Pixel-Based	StarDist	P-value	Pixel-Based	StarDist	P-value	Pixel-Based	StarDist	P-value
All	32	6	<0.0001	22	5	0.0001	27	2	<0.0001
CD3+	17	6	0.0126	12	0	0.0002	35	5	<0.0001
CD4+	14	2	0.0015	10	0	0.0010	16	3	0.0012
CCR5+	17	4	0.0018	11	0	0.0004	15	3	0.0025
CD3+CD4+	5	0	0.0547	2	0	0.4937	11	3	0.0367
CD3+CCR5+	8	1	0.0289	2	0	0.4937	14	2	0.0015
CD4+CCR5+	8	2	0.0872	3	0	0.2405	7	3	0.3109
CD3+CD4+CCR5+	2	0	0.4937	2	0	0.2405	2	0	0.4937

*Number of images (out of 40 images of tissue regions with high cell density) where cells were inaccurately merged or split during automated cell segmentation.

**Number of images (out of 40 images of tissue regions with high abundance of autofluorescent collagen fibers) where autofluorescence was misidentified as cells.

Note: Differences in Cell Merging, Cell Splitting, and Autofluorescence Misidentification between the Pixel-Based method and StarDist method was assess using Fisher's exact tests.

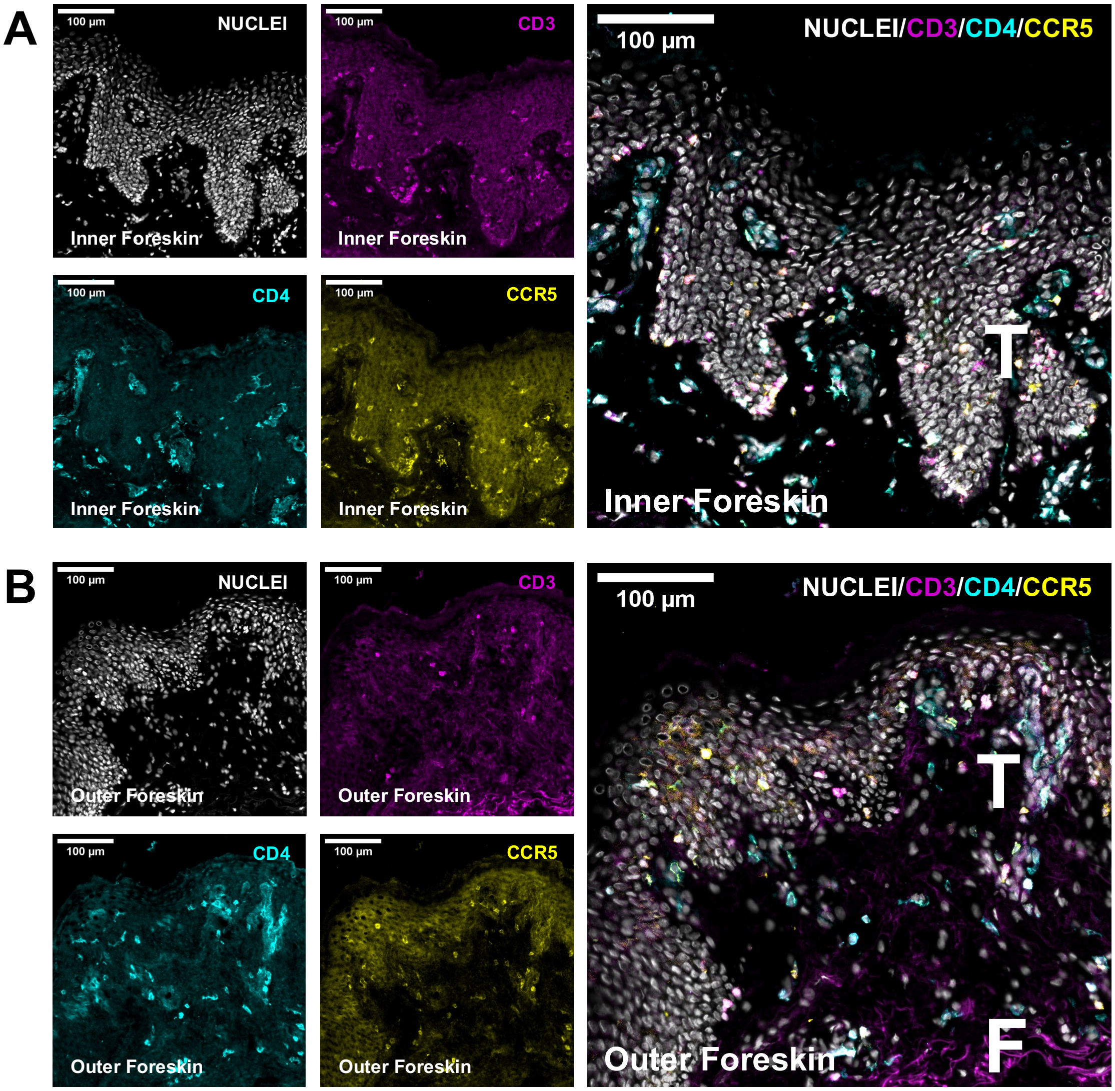

Supplementary Figure 1: Representative images of HIV target cells in foreskin tissue. Foreskin tissue sections were stained with antibodies against CD3, CD4, and CCR5 and with DAPI for nuclei. Whole **(A)** inner and **(B)** outer foreskin tissue sections were imaged at 200x total magnification. Individual staining for CD3, CD4, CCR5, and nuclei is shown on the left while merged composite images from all channels is shown on the right. White letters mark areas containing cell clusters that are likely to contain HIV-susceptible CD4+CCR5+ T cells (marked with "T") and areas with significant autofluorescence (marked with "F").

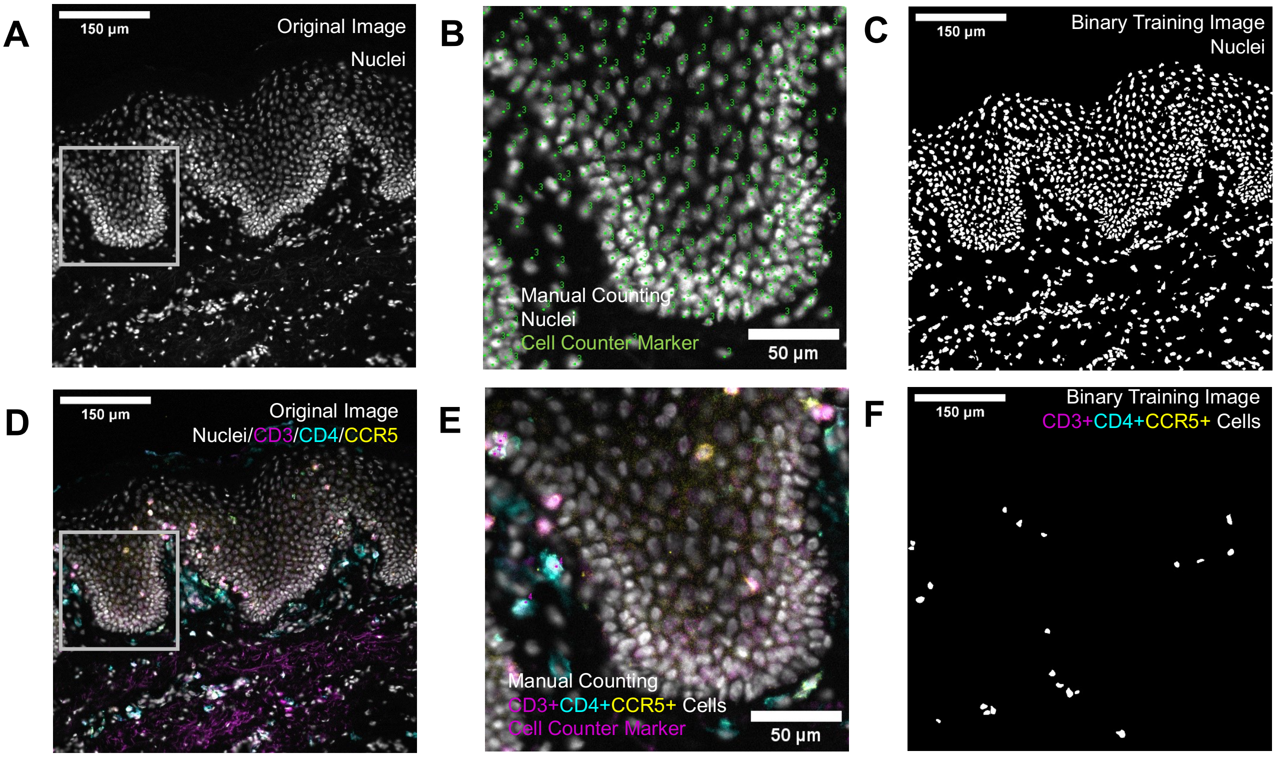

Supplementary Figure 2: Preparation of images for StarDist model training. Workflow of annotating foreskin tissue stained for CD3, CD4, CCR5, and nuclei for StarDist model training. **(A)** Raw image of nuclei staining. **(D)** Composite image of CD3, CD4, CCR5, and nuclei staining to highlight CD4+CCR5+ T cells. Manual cell counting of **(B)** nuclei and **(E)** CD4+CCR5+ T cells using the Cell Counter plugin for Fiji. Tracing of **(C)** nuclei and **(F)** CD4+CCR5+ T cells in the LabKit plugin for Fiji to produce annotated images for training.
